# Phytochemical Composition and Therapeutic Potential of *Ludwigia adscendens*: A Narrative Review

**DOI:** 10.1155/tswj/5359686

**Published:** 2026-03-01

**Authors:** Tasnia Mahmud Easha, Faiza Hamid Jyoti, Farjana Afrin, Mahmuda Nasrin

**Affiliations:** ^1^ Department of Pharmacy, Primeasia University, Dhaka, Bangladesh, primeasia.edu.bd

**Keywords:** ethnomedicinal, *Ludwigia adscendens*, pharmacological, phytochemical, traditional uses

## Abstract

*Ludwigia adscendens* subsp. *diffusa*, an aquatic plant with an extended tradition of use in conventional healthcare, has received growing recognition for its variety of bioactive compounds. Numerous components, notably flavonoids, phenolics, tannins, alkaloids, and terpenoids, have been shown through phytochemical analyses to be responsible for the plant′s pharmacological effects. These compounds are known to have actions such as antioxidant, anti‐inflammatory, antimicrobial, antidiabetic properties, and anticancer effects. Initial phytochemical and pharmacological screenings have indicated therapeutic potential in addressing cardiovascular diseases, diabetes, and microbial infections; however, the current data are still incomplete. A comprehensive chemical characterization and an extensive assessment of its pharmacological effects are essential for enhancing our comprehension of its curative properties. Whereas in vitro and in vivo findings are promising, further investigation into clinical relevance and safety profiles is necessary to confirm *L*. *adscendens* as a potential therapeutic agent.

## 1. Introduction

*Ludwigia adscendens* is a creeping aquatic perennial plant belonging to the Onagraceae family. *Ludwigia* is a vast genus that is found in tropical and subtropical countries like the African continent, Australia, and the Caribbean islands, in addition to temperate places like Europe [1]. With about 82 species distributed across 23 sections, *Ludwigia* is one of the biggest and most varied genera in the Onagraceae family [2]. It is additionally referred to as a floating primrose‐willow or water primrose. Originating from tropical and subtropical areas, it is frequently observed in marshes, ponds, rivers, and paddy fields. Traditionally, it has been employed in folk remedies to treat conditions such as inflammation, skin disorders, and gastrointestinal ailments. Recent research has uncovered the diverse phytochemical profile of this plant, which underscores its therapeutic potential. This aquatic plant has been used traditionally in various regions for its medicinal properties, particularly for its antioxidant, anti‐inflammatory, and antimicrobial activities [3]. Phytochemical studies have revealed the presence of significant bioactive compounds including flavonoids, phenolic acids, and triterpenoids, which contribute to its therapeutic effects.

The plant is an ideal choice to develop medications to treat infections and inflammatory illnesses because it has strong antibacterial and anti‐inflammatory properties. Furthermore, additional research has demonstrated the cytotoxic capability of *L. adscendens*, indicating its potential use in cancer therapy via mechanisms such as inducing apoptosis. In vitro tests and animal models have both demonstrated its anti‐inflammatory qualities, which raises the possibility that it could be used to treat diseases including inflammatory bowel disease and arthritis [4]. The arthritic effect can be lessened by natural chemical elements derived from medicinal plants that interact with and modify the expression of proinflammatory signaling on the inflammation pathway [5]. Furthermore, tests demonstrating the plant′s extract enhanced liver cell viability and decreased oxidative damage indicate that *L. adscendens* possesses hepatoprotective qualities. According to these results, it might be useful in creating therapies for disorders of the liver. Moreover, cytotoxicity investigations have demonstrated that *L. adscendens* has anticancer qualities, as shown by its suppression of cancer cell growth, including carcinomas of the breast and colon cells. The objective of this review is to provide an overview of the current understanding on the phytochemical composition and pharmacological activity of *L. adscendens*, with the goal of shedding illumination on the plant′s potential uses in medicine. We desire to facilitate more research into this historically significant medicinal herb by compiling recent findings.

## 2. Methodology

A literature survey of *L. adscendens* was conducted utilizing various databases, including Elsevier, Chemical Abstracts, ScienceGate, ResearchGate, Google Scholar, and Hindawi with supplementary records obtained from other sources as well. Standard research publications that provided thorough pharmacological and folk medicine viewpoints were chosen. A total of 31 articles, covering the years 1934–2024, were examined to collect pertinent data for a thorough evaluation of the pharmacological and phytochemical properties of *L. adscendens*. A thorough basis for assessing the pharmacological and phytochemical characteristics of *L. adscendens* was established by this methodical approach, which ensured that contemporary as well as traditional findings were included.

## 3. Results and Discussion

### 3.1. Taxonomy and Morphology

The amphibious ascending plant *L. adscendens* is 60 cm long and has branching, floating, oblong stems that reach a height of 4 m. The thick leaves measure 1.25–7.6 cm in length. The white flowers have a faint yellow underneath, and the oblong petals measure around 1.25 cm in length [6]. The axils of the upper leaves bear the long, stalked, solitary, bisexual blooms that grow about 1 cm long. The calyx is tubular, glabrous, and 1.0–1.3 cm long, with five oblong, lanceolate lobes that are each 0.7–0.9 cm long and connected to the ovary. The corolla was composed of five ovoid, round, emarginate petals that were 0.8–2 cm long and 0.8–1.2 cm wide. Ten filaments were present in stamens, arranged in two rings, the outer of which was shorter than the inner [4–6].

### 3.2. Scientific Classification

In accordance with the botanical scheme of Engler, the plant is classified as follows:

Kingdom: Plantae, Division: Spermatophyta, Class: Dicotyledoneae, Order: Myrtales, Family: Onagraceae, Genus: *Ludwigia*, Species: *Ludwigia adscendens.*

### 3.3. Distribution and Propagation

#### 3.3.1. Habit and Habitat

The perennial herb *L. adscendens* is distinguished by its creeping, floating growth habit. Its stems contain spongy, air‐filled tissues known as aerenchyma, which help it float on the water′s surface. The diverse *L. adscendens* plant is adaptable in a variety of water types, including soft and hard water, with a pH level that varies between 6.0 and 7.8. It thrives when given a high‐nutrient substrate and CO_2_ infusion, and it requires ample sunlight and sufficient nutrients to grow healthily [7].

#### 3.3.2. Propagation

Vegetative propagation: The main form of vegetative propagation for *L. adscendens* is dispersion. Its floating stems break readily, and the broken pieces, if they settle in appropriate shallow aquatic settings, can grow roots. The fragments float and are disseminated by water currents because of the plant′s aerenchyma, which are buoyant, spongy tissues [8].

Sexual propagation: Additionally, the plant may germinate by seed, yet this is less prevalent than vegetative reproduction. Insects pollinate the blossoms of *L. adscendens*, resulting in the formation of tiny seeds that can sprout in muddy, humid environments or shallow water [9].

#### 3.3.3. Vernacular Name [9–12]

In different countries and regions, *L. adscendens* is referred to by different colloquial names:

English: Water primrose; Hindi: Kaali Sarson; Bengali: Jolshosh; and Thai: Bua Nam.

#### 3.3.4. Traditional Uses of *L. adscendens*

*L. adscendens* is used for its possible anti‐inflammatory and diuretic effects in an array of traditional systems. It is used frequently to address renal health issues and urinary diseases. Certain regions of humanity, specifically the region of Southeast Asia, eat the fresh leaves and shoots as greens. Because of their considerable dietary value, they are frequently added to soups and salads [13]. In some cultures*, L. adscendens* is frequently included in traditional ceremonies and rites. Many individuals believe that its presence in water bodies contributes to the spiritual qualities of the environment. Rheumatism and other inflammatory diseases are typically treated via *L. adscendens* leaves. It helps cure issues with renal function and urinary tract infections [14]. The herb is utilized in conventional medicine to treat wounds and stomach issues [15].

#### 3.3.5. Ethnomedicinal Uses of *L. adscendens*

The scientific study of the interaction between humans and plants is known as ethnobotany. It covers both contemporary and traditional knowledge of plants used for fuel, agrochemicals, medicine, food, fibers, construction materials, art, cosmetics, dyes, rituals, and magic. A more comprehensive definition also takes into account how people categorize, recognize, and engage with plants, as well as how people and plants interact with one another. Within ethnobotany, there are numerous subfields that concentrate on certain facets of the subject, such as Ethnomedicine, which is the study of traditional medicine, which includes herbal medicines as well as diagnostic and therapeutic procedures. A paste is prepared from the plant′s leaves and applied to the biting site once daily for a period of 2 days to alleviate snack bites. Juice extract is administered to treat dysentery. Plant paste made from leaves is applied topically to treat ailments of the skin. A decoction made from the plant′s leaves is administered to cure fever, dysuria, wheezing, and viral infections [16, 17].

#### 3.3.6. Phytochemical Composition

According to earlier research, the methanol extract of *L. adscendens* contains various chemical groups including gums, glycosides, alkaloids, tannins, flavonoids, saponins, reducing sugars, steroids, and phenolics [18]. Numerous investigations have demonstrated that the ethyl acetate and n‐butanol fractions of various parts contain chemical groups like octyl gallate, 23‐O‐coumaroyl hederagenin‐28‐O‐*β*‐D‐glucopyranoside, quercetin‐3‐O‐glucoside, quercetin 3‐O‐*α* L‐rhamnoside‐2 ^″^‐(4 ^″^‐O‐n‐pentanoyl)‐gallate, and several glycosides [19–25]. Flavonoids (quercitrin, quercetin 3‐O‐rhamnoside, quercetin 3‐O‐galactoside, quercetin 3‐O‐rutinoside, and myricetin 3‐O‐galactoside, kaempferol 3‐O‐glucoside, myricetin 3‐O‐rhamnoside, or myricitrin) and plenty of alkanes were among the multiple phytoconstituents found by a thorough phytochemical analysis [26–28]. Shilpi et al. additionally presented some chemical constituents from *L. adscendens* including betulonic acid, betulin, and betulinic acid, squalene, phytosterols (24R)‐6b‐hydroxy‐stigmasta‐4‐en‐3‐one, (22E,24R)‐6b‐hydroxy stigmasta‐4,22‐dien‐3‐one, ellagic acid derivatives (pteleoellagic acid and 3,3,4‐tri‐O‐methyl ellagic acid), other flavonoids [dihydroquercetin and afzelin (kaempferol 3‐O‐rhamnoside)], protocatechuic acid, and methyl gallate (Table 1) [29].

**Table 1:** Reported phytochemical constituents in *Ludwigia adscendens*.

SL no.	Compound name	Chemical structure	References
1.	Myricitrin	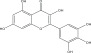	[26–28]
2.	Gallic acid	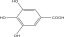	[29]
3.	Methyl gallate	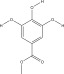	[29]
4.	Quercitrin	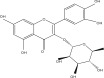	[26–28]
5.	Afzelin	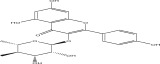	[29]
6.	Protocatechuic acid	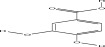	[29]
7.	Quercetin	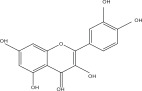	[26–28]
8.	Dihydroquercetin	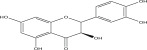	[29]
9.	Squalene	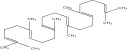	[29]
10.	Pteleoellagic acid	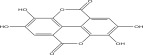	[29]
11.	Betulin	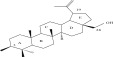	[29]
12.	Betulinic acid	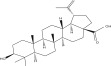	[29]
13.	Betulonic acid	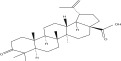	[29]
14.	Hederagenin	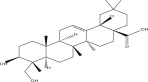	[19–25]
15.	Myricetin‐3‐O‐*α*‐l‐rhamno pyranoside	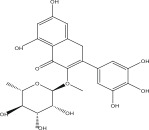	[26–22]
16.	Quercetin 3‐O‐*α*‐l‐rhamnoside‐2 gallate	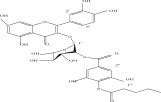	[19–25]
17.	Quercetin‐3‐O‐glucoside	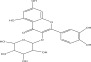	[19–25]
18.	23‐O‐Coumaroyl hederagenin‐28‐O‐*β*‐D‐glucopyranoside	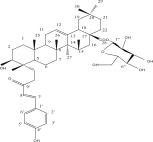	[19–25]
19.	3‐O‐[*β*‐D‐ glucopyranoside‐*α*‐l‐rhamno pyranoside]‐23‐O‐feruloyl‐hederagenin‐28‐O [*α*‐l‐rhamno pyranoside*β*‐D‐glucopyranoside]	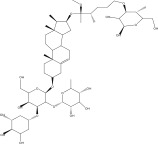	[19–25]
20.	Octyl gallate	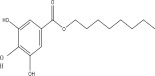	[19–25]
21.	Myricetin 3‐O‐galactoside	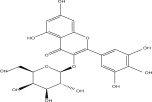	[26–28]
22.	Kaempferol 3‐O‐glucoside	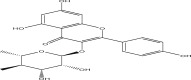	[26–28]
23.	Quercetin 3‐O‐rutinoside	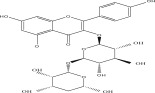	[26–28]
24.	Quercetin 3‐O‐galactoside	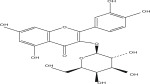	[26–28]
25.	*α*‐d‐heptaglucoside	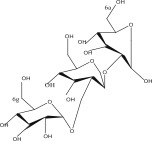	[29]
26.	*α*‐d‐hexaglucoside	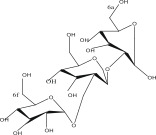	[29]
27.	*α*‐d‐pentaglucoside	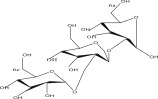	[29]
28.	*α*‐d‐tetraglucoside	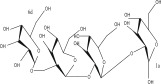	[29]
29.	3,3 ^′^,4 ^′^‐tri‐O methyl ellagic acid	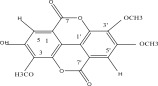	[29]
30.	A mixture of (24R)‐6b‐hydroxy‐stigmast‐4‐en‐3‐one and (22E,24R)‐6b‐hydroxy‐stigmast 4,22‐dien‐3‐one (5a and b)	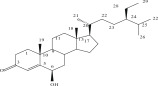	[29]

#### 3.3.7. Pharmacological Studies

##### 3.3.7.1. Antidiabetic Activity.

The *α*‐glucosidase inhibition assay was utilized to evaluate the in vitro antidiabetic potential of *L. adscendens* aerial parts total extract, ethyl acetate fraction, and n‐butanol fraction at various concentrations against acarbose, as it is considered to be the standard antidiabetic drug available. The findings showed that *L. adscendens* ethyl acetate fraction had the largest *α*‐glucosidase enzyme inhibitory effect when compared with n‐butanol fraction and entire extract, with an IC_50_ value of 62.30 *μ*g/mL contrasted to that of acarbose (30.57 *μ*g/mL). Its rich phytochemical composition—especially the presence of bioactive compounds like flavonoids and triterpenoids, which are known to modulate carbohydrate metabolism and inhibit key enzymes involved in glucose absorption—is thought to explain the significant inhibitory activity of the ethyl acetate fraction [21]. In alloxan‐induced diabetic mice, oral administration of methanolic extract of *L. adscendens* (MELA) leaves at 150 and 300 mg/kg led to significant reductions in blood glucose, suggesting antidiabetic potential (Table 2) [18].

**Table 2:** Pharmacological properties of *Ludwigia adscendens*.

Pharmacological properties	Parts of plant	Solvent extract	References
Antidiabetic activity	Aerial parts, whole plant	n‐Butanol, ethyl acetate extract	[18] [21]
Hepatoprotective activity	Aerial parts	n‐Butanol, ethyl acetate extract	[21]
Cytotoxicity activity	Aerial parts	n‐Butanol, ethyl acetate petroleum ether, chloroform, and methanol extract	[7] [18] [25, 30]
Toxicological activity	Whole plant	Methanolic extract	[18]
Antioxidant Activity	Aerial parts, whole plant	Petroleum ether, chloroform, and methanol extract	[7] [29]
Antimicrobial activity	Aerial parts, whole plant	Petroleum ether, chloroform, and methanol extract	[7] [30]
Anti‐inflammatory activity	Whole plant	Methanolic extract	[7]
Antiarthritics activity	Whole plant	Methanolic extract	[7]
Thrombolytic activity	Whole plant	Methanolic extract	[7]
Membrane stabilizing activity	Whole plant	Methanolic extract	[7]
Analgesic activity	Whole plant	Methanolic extract	[18] [31]
Anthelmintic activity	Whole plant	Methanolic extract	[18]
Anticancer activity	Whole plant	Methanolic extract	[18]

##### 3.3.7.2. Hepatoprotective Activity.

The hepatoprotective potential of *L. adscendens* aerial parts was assessed using the MTT test, which measures hepatocyte cell viability. The whole plant extract, as well as its ethyl acetate and n‐butanol fractions, were examined at different concentrations and compared with silymarin, a commonly used reference hepatoprotective drug. The ethyl acetate and n‐butanol fractions showed moderate protective effects, with EC_50_ values of 80.75 and 97.96 *μ*g/mL, respectively, compared with silymarin′s EC_50_ value of 39.64 *μ*g/mL. The observed hepatoprotective benefits are ascribed to the high concentration of antioxidant phytoconstituents, specifically flavonoids and polyphenols, found in these fractions. These chemicals most likely serve an important function in protecting hepatocytes from oxidative damage and promoting cellular integrity (Table 2) [21].

##### 3.3.7.3. Cytotoxic Activity.

One notable bioassay for determining the biological activity of plant extracts at an early stage is the brine shrimp lethality assay. When screening plants, cytotoxicity testing provides crucial details regarding the extract′s possible antitumor and anticancer properties. The LC_50_ value measures the bioactivity of the plant extract, defined as the concentration required to kill 50% of brine shrimp nauplii. The cytotoxic effect of MELA on brine shrimp growth was assessed. Their ethnopharmacological effects may therefore be due to the numerous bioactive compounds present in this plant, such as polyphenols, flavonoids, and tannins [7, 30]. In another study, in the in vitro assay against PC‐3 prostate cancer cells, the total extract, ethyl acetate fraction, and n‐butanol fraction of *L. adscendens* aerial parts were evaluated for cytotoxic activity. Among them, the ethyl acetate fraction showed the most potent effect, with an IC_50_ value of 52.2 *μ*g/mL, suggesting its potential role in inhibiting prostate cancer cell proliferation [25]. The results of this investigation provide trustworthy proof that *L. adscendens* includes possible anticancer chemicals. These substances exhibit promise for additional research to better comprehend and examine their potential for medicinal development (Table 2) [18].

##### 3.3.7.4. Toxicological Studies.

Mice that received MELA leaves orally at dosages of 1000 and 3000 mg/kg showed no signs of mortality, altered behavior, or deformities in their organs; however, a dose of 5000 mg/kg caused death, suggesting that larger concentrations of MELA could be detrimental (Table 2) [18].

##### 3.3.7.5. Antioxidant Activity.

In a dose‐dependent manner, the MELA exhibited the highest DPPH free radical scavenging activity of all the investigated samples. Flavonoids and other polyphenolic components of the extract contributed to reducing DPPH radicals to the nonreactive DPPH‐H form, suggesting that it has substantial antioxidant potential [29]. Another study demonstrated that DPPH is an easy and fast method to measure antioxidant activity since it turns into a violet solution that becomes colorless when reduced by antioxidant molecules. Among all tested samples, MELA exhibited the strongest DPPH radical scavenging activity with an IC_50_ value of 85.76 *μ*g/mL and all samples showed dose‐dependent scavenging effects [Table 2] [7].

##### 3.3.7.6. Antimicrobial Activity.

*L. adscendens* was found to possess antimicrobial properties in five bacteria, namely, *Bacillus cereus*, *Klebsiella pneumoniae*, *Vibrio cholerae*, *Escherichia coli*, and *Streptococcus aureus*, as well as four fungi, *Penicillium chrysogenum, Aspergillus niger*, *Saccharomyces cerevisiae*, and *Mucor hiemalis*. The outcome of their investigation revealed that the MELA exhibited the highest degree of antibacterial activity toward *E. coli* and *B. cereus*, which are responsible for dangerous illnesses such as asthma, pneumonia, indigestion, diarrhea, and infections of the bladder (Table 2) [7, 30].

##### 3.3.7.7. Anti‐Inflammatory Activity.

A crucial method for assessing the potential therapeutic advantages of various plants is the in vitro anti‐inflammatory evaluation of plant extracts. The egg albumin denaturation test is frequently utilized to evaluate the anti‐inflammatory properties of plant extracts. If a plant extract demonstrates the ability to inhibit the denaturation of egg albumin, it may be regarded as possessing anti‐inflammatory properties. The outcome derived from the egg albumin denaturation assay serves as a valuable tool for screening plant extracts for their anti‐inflammatory properties. Results showed that *L. adscendens* has the highest egg albumin denaturation effect at concentrations of 1000 and 500 *μ*g/mL compared with the standard acetylsalicylic acid, a standard drug that is often used as an anti‐inflammatory medication (Table 2) [7]

##### 3.3.7.8. Antiarthritics Activity.

The impact on protein denaturation inhibitions is demonstrated in certain research. The results indicated that protein denaturation was inhibited by both the MELA and standard drug (diclofenac‐sodium) in a concentration‐dependent manner throughout the entire concentration range of 62.5–1000 *μ*g/mL. The crude extract exhibited a protein denaturation inhibitory activity of 98.195 at 1000 *μ*g/mL, which was comparable to the activity of diclofenac‐sodium (94.59% at 100 *μ*g/mL). In addition, the antiarthritic activity of plant extract is frequently examined in vitro by denaturing bovine serum albumin (BSA). In order to accomplish this, researchers investigate the influence of herbal compounds on the stability of BSA, a protein that shares numerous structural and functional similarities with human serum albumin (HSA). In the assay, protein denaturation is quantified as a result of exposure to extremes in pH or ionic strength, thermal or chemical treatment, or both. An anti‐inflammatory substance or an antiarthritic chemical may mitigate or prevent denaturation. The antiarthritic properties of the plant extract are attributed to their bioactive components, which inhibit inflammatory pathways, scavenge free radicals, and modulate the immune system, respectively (Table 2) [7].

##### 3.3.7.9. Thrombolytic Activity.

A controlled laboratory setting is used to evaluate the potential of MELA to dissolve blood clots through the use of a thrombolysis test. Blood cells and proteins form a clot to prevent additional hemorrhaging when the body is injured. Nevertheless, heart attacks and strokes, which are potentially catastrophic, may result from excessive clotting. Bhuiyan et al. demonstrated that the percentage of thrombus lysis of plant extract is significantly higher than that of standard streptokinase (Table 2) [7].

##### 3.3.7.10. Membrane Stabilizing Activity.

The membrane stabilizing test is a widely used method for assessing the medicinal properties of plants. This experiment evaluates the efficacy of plant extract in safeguarding against membrane lysis and injury, which are two of the most prevalent side effects of pathological conditions, such as oxidative stress and inflammation. The test involves the application of osmotic stress to a lipid membrane and the subsequent measurement of the membrane′s stabilization in the presence of the plant extract. Results indicated the MELA exhibited a substantial membrane stabilizing activity with a difference of 63.14% from the conventional drug (Table 2) [7].

##### 3.3.7.11. Analgesic Activity.

A well‐known way to test how effective different pain‐relief drugs are, which work on the spinal cord, is by looking at how mice respond to heat [31]. The analgesic effect of MELA was tested in Swiss albino mice weighing 22–25 g at 250 and 500 mg/kg orally b.w. using hot plate, formalin‐induced, and acetic acid writhing method. The MELA was shown to reduce the incidence of acetic acid–induced writhing in mice when given at higher doses. The findings of the pharmacological test signify that the methanol extract possesses both central and peripheral antinociceptive properties given the antinociceptive effects seen in it during a variety of tests. As a result, it may be regarded as a more effective therapeutic option than the NSAIDs and analgesics already on the market (Table 2) [18].

##### 3.3.7.12. Anthelmintic Activity.

The testing was carried out on mature earthworms due to their physiological and physical resemblance to intestinal roundworm parasites in humans. Earthworms are widely used for initial in vitro evaluation of anthelmintic substances due to their easy availability. The MELA leaves function as anthelmintic activity in vitro against adult *Haemonchus contortus*. In order to evaluate the anthelmintic effectiveness of fresh leaf juice, earthworms were subjected to several amounts of the liquid (5, 10, 20, 50, and 100 mg/mL). Twenty worms received each treatment at a regulated temperature of 35^°^C ± 1^°^C. Each therapy was in three copies. Because the anthelmintic treatment reduced worm movement, it was advantageous. The times for paralysis, total inaction, and death were noted at intervals of 0, 1, 2, and 4 h. These findings align with the anthelmintic effect observed with standard medication albendazole (Table 2) [18].

##### 3.3.7.13. Anticancer Activity.

The anticancer activity was evaluated in HeLa cells cultured in DMEM supplemented with 10% FBS, 1% penicillin–streptomycin, and 0.2% gentamycin, seeded in 96‐well plates, treated with 25 *μ*L of filtered MELA, and assessed after 48 h using the CellTiter 96 Nonradioactive Cell Proliferation Assay (Promega, United States) in duplicate. The plant material was standardized according to established procedures, and the anticancer potential of the standardized alcoholic extract was investigated using HeLa cell lines. The findings provide evidence that MELA contains bioactive compounds with potential anticolon cancer properties, warranting further investigation for therapeutic development (Table 2) [18].

### 3.4. Conclusion

*L. adscendens* is a valuable herb that is utilized in conventional medical practice for the treatment of various ailments and maintenance of overall health and well‐being. Comprehensive pharmacological research has established the scientific foundation for the medicinal applications of *L. adscendens* in the management of numerous illnesses. Although many pharmacological studies have been conducted on this plant, very few preliminary phytochemical studies have been documented. Thus far, flavonoids, terpenes, triterpenoids, phenols, tannins, alkaloids, and carbohydrates have been identified as the main constituents. This review compiles data on *L. adscendens*, emphasizing its pharmacological, nutritional, and antibacterial properties along with its chemical composition. The study finishes by illustrating the importance of additional research on this plant, due to its considerable potential for the future.

## Funding

No funding was received for this manuscript.

## Disclosure

The authors hereby declare that the work presented in this article is original and that any liability for claims relating to the content of this article will be borne by them.

## Conflicts of Interest

The authors declare no conflicts of interest.

## Data Availability

The data that support the findings of this study are available on request from the corresponding author. The data are not publicly available due to privacy or ethical restrictions.
